# Mitigation of sterigmatocystin exposure in cattle by difructose anhydride III feed supplementation and detection of urinary sterigmatocystin and serum amyloid A concentrations

**DOI:** 10.5194/aab-64-257-2021

**Published:** 2021-06-16

**Authors:** Naoya Sasazaki, Seiich Uno, Emiko Kokushi, Katsuki Toda, Hiroshi Hasunuma, Daisaku Matsumoto, Ayaka Miyashita, Osamu Yamato, Hiroaki Okawa, Masayuki Ohtani, Johanna Fink-Gremmels, Masayasu Taniguchi, Mitsuhiro Takagi

**Affiliations:** 1 Joint Graduate School of Veterinary Sciences, Yamaguchi University, Yamaguchi 753-8515, Japan; 2 Shepherd Central Livestock Clinic, Kagoshima 899-1611, Japan; 3 Faculty of Fisheries, Kagoshima University, Kagoshima 890-0056, Japan; 4 Shiiba Village Office Livestock Clinic, Shiiba 883-1601, Japan; 5 Joint Faculty of Veterinary Medicine, Kagoshima University, Kagoshima 890-0062, Japan; 6 Guardian Co. Ltd., Kagoshima 890-0033, Japan; 7 Nippon Beet Sugar Manufacturing Co., Ltd., Obihiro 080-0835, Japan; 8 Faculty of Veterinary Medicine, Utrecht University, Yalelaan 104, Utrecht, the Netherlands

## Abstract

We evaluated the effects of supplementing cattle feed with difructose anhydride III (DFA III) by measuring urinary sterigmatocystin (STC) concentrations using 20 Japanese Black cattle aged 9–10 months from one herd. DFA III was supplemented for 2 weeks for 10 animals, and non-treated animals served as controls. The natural STC concentration in the dietary feed was 0.06 $\mathrm{mg}\;{\mathrm{kg}}^{-1}$ (mixture of roughage and concentrate) at the beginning of the study (Day 0). The urine STC concentration was measured using liquid chromatography with tandem mass spectrometry 1 d prior to DFA III administration, 9 and 14 d thereafter, and 9 d following supplementation cessation, concomitant with the measurement of serum amyloid A (SAA). The number of heifers in which STC was detected in the urine was low (10 %) in the DFA III group compared to that (60 %) in the control group on Day 9. After 9 d following supplementation cessation (Day 23), STC concentrations were significantly lower (P=0.032) in the DFA III group than in the control group, although there was no difference in the number of heifers in which urinary STC was detected or in SAA concentrations between the two groups. Our findings demonstrate the effect of DFA III on reducing the urinary concentration of STC in Japanese Black cattle.

## 1 Introduction

The contamination of agricultural commodities with mycotoxins is a major worldwide challenge in agriculture and livestock production (Fink-Gremmels, 2008). Recently, more attention has been paid to the impact of mycotoxins, as global warming has exacerbated exposure and thereby enhanced the risk for harmful effects on both human and animal health (Liew and Mohd-Redzwan, 2018; Raduly et al., 2020). The contamination of food and feed chains with *Aspergillus*-derived mycotoxins poses a significant global challenge (Raduly et al., 2020). Sterigmatocystin (STC) is generally considered an *Aspergillus*-derived mycotoxin but can be produced by some fungi of the genus *Penicillium* as well. This mycotoxin is considered the end product of a biosynthetic pathway in some fungal species, such as *Aspergillus versicolor* and *Aspergillus nidulans*, and is also a well-known precursor for the synthesis of aflatoxin (AF) B1 (Hsieh et al., 1973; Wilkinson et al., 2004; Versilovskis and de Saeger, 2010). Currently, there is no consensus on the maximum tolerable limit of STC in food or feed; the European Food Safety Authority (EFSA) classified STC as a possible human carcinogen (Group 2B). This classification was based on research data indicating that STC has carcinogenic, mutagenic, neurotoxic, immunogenic, and estrogenic effects in vitro and in vivo (EFSA Panel on Contaminants in the Food Chain, 2013; Kusunoki et al., 2011). Several incidences of STC contamination in food and feed (e.g., grains, grain-based products, maize, and rice) have been also reported from Japan (Kobayashi et al., 2018; Nomura et al., 2018; Kobayashi et al., 2019; Yoshinari et al., 2019). In our previous study, in which STC contamination levels in cattle feed were monitored, we measured urinary STC concentrations using liquid chromatography with tandem mass spectrometry (LC-MS/MS) (Fushimi et al., 2014b) and found that monitoring urinary STC levels helps to evaluate the contamination level in cattle herds. Additionally, we have also assessed the efficiency of mycotoxin detoxification supplements in dietary feed to impair the intestinal adsorption of mycotoxins (Takagi et al., 2011; Hasunuma et al., 2012; Fushimi et al., 2014a).

Several approaches for the prevention of *Aspergillus*-derived mycotoxins in both preharvest and postharvest stages have been reported, such as appropriate agricultural practice and crop management, the introduction of a non-toxigenic antagonistic fungal strain into the field prior to harvest, adequate storage management, and the application of fungicidal agents and other protective silage additives at the postharvest stage. As these measures are only partly effective, it has become common practice to apply adsorbing agents (denoted as mycotoxin adsorbents, MAs), such as mineral clays, probiotics and prebiotics (particularly yeast), yeast cell fractions, lactic acid bacteria, and mycotoxin-degrading enzymes, to animal feed (Sabater-Vilar et al., 2007; Kutz et al., 2009; Awad et al., 2010; Wambacq et al., 2016). Generally, MAs consist of a mixture of a mineral clay carrier, yeast cell wall preparations, and in some cases enzymes or living microorganisms (probiotics) that can adsorb and detoxify mycotoxins (Liew and Mohd-Redzwan, 2018; Vila-Donat et al., 2018). Previously, we have not observed any decrease in STC concentration in the urine after adding a mineral clay carrier mixed with enzymes as a detoxification supplement in cattle feed (Fushimi et al., 2014b), although clay is generally known to be an effective detoxification agent against AFs (Raduly et al., 2020).

In addition, considering the dietary exposure of animals to complex mixtures of mycotoxins inherent to a professional mixed diet, there is a growing interest in the health-promoting benefits of prebiotics and non-digestible oligosaccharides, such as mannan-oligosaccharides (Heinrichs et al., 2003; Franklin et al., 2005), fructooligosaccharides (Donovan et al., 2002), and lactulose (Fleige et al., 2007), to reduce the incidence of diseases in animals (Fleige et al., 2009). In particular, oligosaccharides have been shown to interact with intestinal epithelial cells, decreasing their inflammatory reaction to dietary challenges, such as mycotoxins or physiological stressors including heat stress, by upregulating tight-junction protein expression and modulating the intestinal immune responses (Akbari et al., 2015, 2017a, b; Varasteh et al., 2018). We previously reported the benefits of difructose anhydride III (DFA III), a unique non-digestible disaccharide present in commercial roasted chicory and manufactured from inulin through microbial fermentation (Sato et al., 2007; Teramura et al., 2015), which seems to have these properties. When DFA III was used as an anti-mycotoxin supplement, the concentration of zearalenone (ZEN) in the urine of cattle declined, as in indication of reduced systemic bioavailability in the presence of DFA III. However, the exact mechanism, which is likely related to the protective effect of DFA III on the intestinal barrier, has to be elucidated (Toda et al., 2018). Based on these findings, we hypothesized that DFA III could be an effective anti-mycotoxin supplement against STC. Hence, the aim of this study was to evaluate the effects of DFA III supplementation on the concentration of STC in the urine as an indicator of the impact of DFA III on mycotoxin absorption. In addition, serum amyloid A (SAA), one of the most reliable positive acute-phase proteins mainly produced by the liver and other tissues including the intestines (Berg et al., 2011; Zhang et al., 2018), was also monitored to assess inflammation status and its relationship with changes in STC concentrations.

## 2 Materials and methods

All experiments were conducted according to the guidelines and regulations for the protection of experimental animals and guidelines stipulated by Yamaguchi University, Japan (no. 40, 1995; approved on 27 March 2017).

### 2.1 Chemicals and solvents

STC was purchased from MP Biomedicals (Heidelberg, Germany). Stock solutions of 1 $µg\;{\mathrm{mL}}^{-1}$ STC in acetonitrile were stored in the dark at 4 ${}^{\circ }C$. High-performance liquid chromatography (HPLC)-grade methanol was purchased from Wako Pure Chemical Industries, Ltd. (Osaka, Japan). A β-Glucuronidase/arylsulfatase solution was purchased from Merck (Darmstadt, Germany). Sodium acetate was purchased from Kanto Chemical Co., Inc. (Tokyo, Japan). Tris was purchased from Nacalai Tesque, Inc. (Kyoto, Japan).

### 2.2 Japanese Black cattle herds and sample collection

The details of the animals and collected serum and urine samples used in the present study were as described in our previous study (Toda et al., 2018). In brief, 20 Japanese Black heifers (10 months-old, 250–300 kg) from one beef herd raised in Kagoshima Prefecture, Japan, were included in this experiment. The herd consisted of 500 beef cattle fed purchased concentrates and rice straw. The herd of beef cattle in this study was divided over 10 barns, each containing 2 beef cattle, and a total of 20 beef cattle were sampled in the order in which they entered. Roughage and concentrate were stored at ambient temperature in feed sheds and silos, respectively. The STC level in the dietary feed of the herd was first measured before beginning the experiment using LC-MS/MS, as previously reported (Fushimi et al., 2014b). In brief, representative samples of stored straw (2 g) and concentrate (10 g) were homogenized and chopped into small pieces. To each sample, 20 mL of 84 % acetonitrile was added, shaken for 1 h, and centrifuged for 10 min at 500×g at room temperature. The supernatant (10 mL) was loaded into a MultiSep 226 AflaZon+ multifunctional column (Romer Labs, Union, MO, USA). A total of 1 mL of the eluent was mixed with 1 mL of acetic acid (1+100) and centrifuged for 5 min at 500×g, and 10 µL of the supernatant was injected into an API3200 LC-MS/MS system (Applied Biosystems, Foster City, CA, USA) equipped with an electrospray ionization (ESI) interface and a Prominence HPLC System (Shimadzu Corp., Kyoto, Japan). All conditions of the LC-MS/MS system were the same as previously described (Fushimi et al., 2014b). The detection limit for each analyte was set as 0.01 $\mathrm{mg}\;{\mathrm{kg}}^{-1}$, and the mean STC recovery rates were 90.5 %–93.5 %. The mean concentration of STC in the mixture of roughage and concentrate fed to the heifers was found to be 0.06 $\mathrm{mg}\;{\mathrm{kg}}^{-1}$.

**Figure 1:**
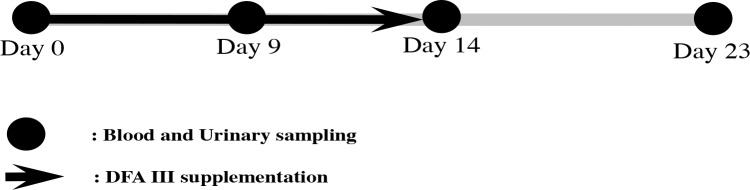
Experimental protocol for DFA III supplementation in dietary feed and sampling of blood and urine.

Heifers were selected from the experimental herd and divided into two groups differing in feed supplementation as follows: the DFA III group (n=10) was fed 40 g of DFA III per day (20 g twice) mixed with concentrate, while the control group (n=10) was fed a DFA III-free diet. As a method of allocation, 10 random numbers were generated by a computer: five barns were randomly allocated to the DFA III supplementation group, and five barns were allocated to the control group. The DFA III dose used herein is the dose recommended for the prevention of hypocalcemia in dairy cows (Sato et al., 2007; Teramura et al., 2015). A total of 2 h after the cattle were fed in the morning, urine samples were collected by massaging the pudenda, and blood samples were collected from the jugular veins into silicon-coated tubes. Sampling was performed at the start of DFA III supplementation (Day 0), on Day 9 and Day 14 (i.e. the last day of DFA III supplementation), and on the last day of the experimental period (Day 23), as shown in Fig. 1. All samples were immediately placed in a cooler containing dry ice, also protecting it from UV light, and immediately transported to the laboratory. All urine and blood samples were centrifuged at 1000 and 2000×g, respectively, for 10 min at room temperature and then frozen at -30 ${}^{\circ }C$ until STC and SAA analyses. The primary outcome of this study was the STC value at Day 23. The secondary outcomes were the STC and SAA values at each measurement (Days 0, 9, 14) and the STC detectable frequency.

### 2.3 Urine sample analysis

First, STC concentrations were determined with LC-MS/MS using an API 2000 system (Applied Biosystems) equipped with an ESI interface and an HPLC system (Agilent 1200 Series; Agilent Technologies, Santa Clara, CA, USA) as previously described (Fushimi et al., 2014b). Briefly, all urine samples (0.5 mL) were mixed with 3 mL of 50 mM ammonium acetate buffer (pH 4.8) and 10 µL of glucuronidase/arylsulfatase solution and incubated for 12 h at 37 ${}^{\circ }C$. Following incubation, the solution was loaded into a C18 solid-phase extraction column (Strata; Phenomenex, Torrance, CA, USA) preconditioned with 3 mL of MeOH and 2 mL of tris buffer, followed by the addition of 2 mL of tris buffer and 3 mL of 40 % MeOH. After elution with approximately 1 mL of 80 % MeOH, the volume was adjusted to exactly 1 mL. Next, 20 µL of the solution was injected into the LC-MS/MS system. Chromatographic separation was performed on an Inertsil ODS-3 column (4.6i.d.×100 mm, 5 µm; GL Sciences, Tokyo, Japan) at 40 ${}^{\circ }C$. A mobile phase consisting of methanol/water/acetic acid (97:3:0.01,v:v:v), was used (200 $µL\;\min^{-1}$) to separate the analyte in the isocratic mode. Measurements were performed for 15 min. The conditions of measurements with LC-MDS/MS were the same as those in our previous study (Fushimi et al., 2014b). The limit of detection (LoD) was 0.2 $\mathrm{ng}\;{\mathrm{mL}}^{-1}$. The concentration of urinary creatinine was determined using a commercial kit (Sikarikit-S CRE; Kanto Chemical Co., Inc.) according to the manufacturer's instructions and assessed using a 7700 Clinical Analyzer (Hitachi High-Tech, Tokyo, Japan). STC concentrations in the urine were expressed as a ratio to creatinine (pg STC ${\mathrm{mg}}^{-1}$ creatinine). In urine samples with no peaks using LC/MS/MS analysis, the concentration was set to zero (pg STC ${\mathrm{mg}}^{-1}$ creatinine).

### 2.4 Measurement of serum amyloid A concentration

All SAA concentrations in the collected samples on Days 0, 9, and 14 were measured using a Pentra C200 automated biochemical analyzer (HORIBA ABX SAS, Montpellier, France) with an SAA reagent specialized for animal serum or plasma (VET-SAA “Eiken” reagent; Eiken Chemical Co., Ltd., Tokyo, Japan). In addition, the concentration of SAA was calculated based on a standard curve made by a VET-SAA calibrator Set (Eiken Chemical Co., Ltd.). Following this, the concentrations of SAA were compared both between and within the groups. In addition, the numbers of heifers in which the concentration of SAA decreased in each sampling period were compared between the two groups.

### 2.5 Statistical analysis

The sample size of this study was selected based on our previous report (Fushimi et al., 2014b). Since this is a pilot study, statistical power was calculated using post-hoc power analysis. All STC and SAA concentrations are expressed as means, standard errors of the mean (SEMs), median, and interquartile range (IQR). For STC value comparison on Day 23 between groups, which is the primary evaluation parameter, the Mann–Whitney U test was used as an ordinal scale in which zero was substituted for the measured value below the lower limit of detection. For the secondary evaluation parameter, the summary statistics of the STC and SAA values at each measurement time point, as well as STC detection frequency, were calculated. For these items, comparison tests were not performed due to difficulties in power and sample size. A p value<0.05 was considered statistically significant. All statistical analyses were performed using Bell Curve for Excel software (Social Survey Research Information Co., Ltd., Tokyo, Japan), and power analyses were performed using G*Power 3.1.9 (Franz Faul, Universität Kiel, Germany).

## 3 Results

During the study period, no significant clinical differences were observed between the DFA III and control groups.

### 3.1 Concentration of sterigmatocystin with or without difructose anhydride III supplementation

**Figure 2:**
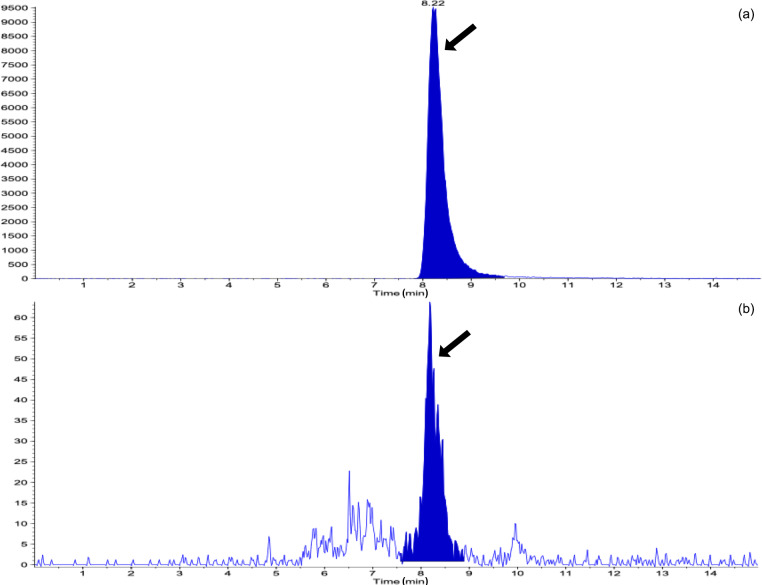
Representative liquid chromatography–tandem mass spectrometry chromatograms for **(a)** the sterigmatocystin (STC) standard (1 $\mathrm{ng}\;{\mathrm{mL}}^{-1}$). **(b)** A clear peak of STC contamination in the urine sample (arrow). The horizontal axis represents time (min).

Table 1 shows the results of the analysis of the concentrations of STC in the urine and the number of heifers in which the STC concentration was above the limit of detection (LoD) during the study period, with or without DFA III supplementation. The STC concentrations in the urine on Day 0 showed that both groups exhibited almost the same level of STC contamination. The number of heifers in which STC was quantifiable in the urine was 1 (10 %) in the DFA III group as compared to six (60 %) in the control group on Day 9. On Day 14, however, no STC was detected in urine samples from either group, possibly due to low-level STC-contaminated parts within the same lot of feed (straw) eaten prior to sampling. A total of 9 d after terminating supplementation (Day 23), STC concentration was significantly lower (P=0.032,power=0.741) in the DFA III group than in the control group, although there was no difference in the number of heifers in which urinary STC was detected between the two groups. Representative chromatograms from the LC-MS/MS assay are shown in Fig. 2.

**Table 1:** Results of the analysis of the number of heifers in which the concentration of STC was above the limit of detection (LoD) during the study period, as well as the concentration of STC (measured as pg ${\mathrm{mg}}^{-1}$ creatinine) in the urine (mean±standard error of the mean, SEM) with or without DFA III supplementation.

	Day 0	Day 9	Day 14	Day 23
DFA III (n=10)				
No. of heifers (%)	3 (30 %)	1 (10 %)	0 (0 %)	5 (50 %)
Concentration${}^{b}$				
Mean±SEM	14.3±8.0	10.5	0	$16.8\pm 6.3^{a}$
Median value	0	0	0	10.3
Interquartile range	21.5	0	0	26.4
Control (n=10)				
No. of heifers (%)	2 (20 %)	6 (60 %)	0 (0 %)	7 (70 %)
Concentration				
Mean±SEM	10.9±7.6	87.1±27.3	0	$67.5\pm 16.8^{a}$
Median value	0	95.6	0	74.2
Interquartile range	0	141.0	0	79.1

${}^{a}$ p=0.032. ${}^{b}$ Concentration in urine samples with no peaks using LC/MS/MS analysis was set as 0 (pg STC ${\mathrm{mg}}^{-1}$) creatinine in the present study. STC stands for sterigmatocystin. DFA III stands for difructose anhydride III.

### 3.2 Concentration of serum amyloid A with or without difructose anhydride III supplementation

Individual cattle from which all samples could not be obtained three times were omitted from these measurements (DFA III group: n=1; control group n=2). The concentrations of SAA ($\mathrm{mg}\;L^{-1}$, mean±SEM) in the DFA III group (n=9) on Days 0, 9, and 14 during the experimental period were 11.8±3.1,5.8±1.2, and 8.1±2.3, respectively, whereas those in the control group (n=8) were 3.8±0.8,3.2±1.3, and 2.2±0.3, respectively, with no significant differences among the sampling days within each group. In addition, no differences were observed in the numbers of heifers in which the concentration of SAA decreased among the sampling periods, i.e., from Day 0 to Day 9 (DFA III: 8 of 9, control: 5 of 8) and from Day 0 to Day 14 (DFA III: 4 of 9, control: 1 of 8).

## 4 Discussion

We have previously reported the benefits of measuring urinary mycotoxin concentrations in cattle to assess the rate of exposure, using zearalenone and STC as examples (Takagi et al., 2011; Fushimi et al., 2014b). We could also show that this approach can be used to assess the efficacy of commercially available MA (Fushimi et al., 2014a). Therefore, considering the desirable effect of DFA III as a feed supplement in reducing the concentration of ZEN in the urine and the hypothesis that this effect was related to the positive effect of DFA III on intestinal barrier functions, we hypothesized that DFA III might also be effective against STC.

Rice straw is considered one of the most important roughages used in the production of beef cattle in Japan. Generally, STC is a major mycotoxin produced in rice. However, the harmful or chronic effects of STC on cattle are not well understood, and there are no regulations or control measures regarding this toxin in Japan. As pointed out in a recent review by Vila-Donat et al. (2018), it is important to assess anti-mycotoxin agents by assessing the feed with natural contaminants using real-life scenarios, paying attention to their efficacy and safety, as well as their potential for interactions with critical nutrients, such as vitamins and minerals. Clay is a widely studied agent used in mycotoxin detoxification, especially in reducing the toxicity of AFs (Raduly et al., 2020). STC has a molecular structure rather similar to that of AFs and acts as an intermediate in the biosynthetic pathway of AFs (EFSA Panel on Contaminants in the Food Chain, 2013). Therefore, it is assumed that clay-based MAs might show comparable effects against STC. However, no experimental studies to support this hypothesis have been performed and negative results were found with a clay-based commercial product in one of our previous studies (Fushimi et al., 2014b). Therefore, in the current study, we monitored the concentration of STC in the urine of cattle to assess the beneficial effects of adding DFA III as a feed supplement. DFA III is currently used as a prebiotic agent and in cases of hypocalcemia in cattle, due to its positive effect on calcium absorption (Teramura et al., 2015). Our results suggest that DFA III might effectively prevent the adsorption of STC in the intestines of cattle, although the results could not be substantiated by statistical analysis due to the limited number of samples. Natural contaminated materials were used in the current study, and at this rather low exposure rate, STC concentrations in urine were not quantifiable in all samples.

When STC is ingested, it is metabolized in the liver and lungs by various cytochrome P450 enzymes into different hydroxymetabolites, and then both conjugated parent STC and its hydroxylated metabolites are excreted via bile and urine (EFSA Panel on Contaminants in the Food Chain, 2013). In a previous report, we indicated that STC is extensively conjugated in the liver, presumably to glucuronic acid, since only trace amounts of free mycotoxin could be detected in the urine (Fushimi et al., 2014b). In this study, however, although the ratio of parent STC reaching systemic circulation remains unknown, our results reconfirmed that even at low natural concentrations of STC (0.06 $\mathrm{mg}\;{\mathrm{kg}}^{-1}$ dry matter) detection in the urine of cattle is possible. The hydroxymetabolites could not be identified in this study, partly due to a lack of reference data. However, forthcoming studies should at least aim to capture glucuronidated STC excreted in urine by applying appropriate sample preparation steps.

Most commercial MAs act either by binding mycotoxins on their surfaces (adsorption) or by degrading or transforming them into less toxic metabolites (biotransformation) (Fleige et al., 2007). We previously reported that the use of a commercial MA (mixed adsorption and biotransformation agents) in cattle significantly reduced the concentration of ZEN in the urine but failed to reduce the levels of STC in the urine. As we assume that DFA III reduces the bioavailability and subsequently the measurable concentration in urine by a different mechanism, we decided to assess the effect of DFA III on STC, which structurally differs from ZEN. Although the number of results in this study is limited, as we used a naturally contaminated diet, results provide a strong indication that DFA III affects the systemic availability and hence the urinary concentration of STC. These results are of importance, as they indicate that DFA III acts differently from the clays used for aflatoxin adsorption. In line with the extensive data on the beneficial effects of non-digestible oligosaccharides on intestinal functions and barrier integrity, DFA III might have a comparable effect. This is supported by the current finding that next to ZEN, DFA III impacted the systemic availability of a structurally entirely different mycotoxin, such as STC.

The intestinal tract is the first organ susceptible to contamination by mycotoxins. It has been suggested that the intestinal barrier plays a crucial role in limiting the absorption of mycotoxins and that exposure to mycotoxins causes inflammatory responses, epithelial cell death, and tight-junction damage in the intestine, leading to a disruption of intestinal barrier function (Liew and Mohd-Redzwan, 2018; Akbari et al., 2015, 2017a, b; Zhai et al., 2019). Moreover, disruption of the intestinal barrier might enhance the translocation of bacterial toxins from the intestine, inducing systemic inflammation (Rao, 2009; Zhai et al., 2019). To assess the level of systemic inflammation in cattle, SAA is often used as a biomarker, as its serum concentration increases in response to various inflammatory stimuli, such as infections, trauma, and toxicity (Berg et al., 2011). However, our results showed that DFA III might not affect the concentration of SAA. This finding might be attributed to the rather low STC concentration in feed (0.06 $\mathrm{mg}\;{\mathrm{kg}}^{-1}$), which might be below the level that causes a systemic inflammatory response, or to the rather short half-life of SAA. Significant changes in SAA levels in the blood are primarily seen at the onset of inflammation, and hence this initial effect might have been overlooked by the current experimental protocol, as the first sampling date was after 9 d of exposure.

## 5 Conclusion

Taken together, the results of this study support the hypothesis that oligosaccharides, such as DFA III, which are now widely being used as prebiotics, can be successfully used as mycotoxin-mitigating substances. The benefit of this approach is a combined effect on adsorption, here measured by declining urinary STC excretion as an indicator of the internal dose, and the known beneficial effects of the prebiotic DFA III on intestinal barrier integrity and gut health. Further field studies with a larger sample size are warranted to establish a database for the assessment of DFA III as a dietary supplement to reduce the absorption of mycotoxins in cattle and other animals. It is also important to further consider the systemic bioavailability of STC in ruminating cattle to improve our understanding of STC contamination and co-contamination with other mycotoxins and to assess the potential risk to cattle health.

## Data Availability

No data sets were used in this article.
